# Psoriasis and Atherosclerosis—Skin, Joints, and Cardiovascular Story of Two Plaques in Relation to the Treatment with Biologics

**DOI:** 10.3390/ijms221910402

**Published:** 2021-09-27

**Authors:** Karina Wierzbowska-Drabik, Aleksandra Lesiak, Małgorzata Skibińska, Michał Niedźwiedź, Jarosław D. Kasprzak, Joanna Narbutt

**Affiliations:** 1 Department of Cardiology, Medical University of Lodz, 91-347 Lodz, Poland; wierzbowska@ptkardio.pl (K.W.-D.); kasprzak@ptkardio.pl (J.D.K.); 2 Department of Dermatology, Pediatric Dermatology and Oncology, Medical University of Lodz, 91-347 Lodz, Poland; aleksandra.lesiak@umed.lodz.pl (A.L.); michal.niedzwiedz@stud.umed.lodz.pl (M.N.); joanna.narbutt@umed.lodz.pl (J.N.)

**Keywords:** psoriasis, psoriatic arthritis, atherosclerosis, heart muscle function, biologic treatment, TNF alfa inhibitors, anti-IL12/23, anti-IL17

## Abstract

It is known that both psoriasis (PSO) limited to the skin and psoriatic arthritis (PSA) increase the risk of cardiovascular complications and atherosclerosis progression by inducing systemic inflammatory response. In recent decades, the introduction of biological medications directed initially against TNF-α and, later, different targets in the inflammatory cascade brought a significant breakthrough in the efficacy of PSO/PSA treatment. In this review, we present and discuss the most recent findings related to the interplay between the genetics and immunology mechanisms involved in PSO and PSA, atherosclerosis and the development of cardiac dysfunction, as well as the current PSO/PSA treatment in view of cardiovascular safety and prognosis.

## 1. Introduction and Methods

Both psoriasis (PSO) and psoriatic arthritis (PSA) are connected to chronic systemic inflammation which also plays a key role in the development of atherosclerosis (Ath) and its cardiovascular complications resulting in myocardial infarctions and strokes. The prevalence of left ventricular hypertrophy and progression to symptomatic heart failure with preserved ejection fraction is significantly increased in both PSO and PSA [1,2]. Beyond the impact of inflammation, the increased prevalence of traditional cardiovascular risk factors is observed in both entities. This may lead to an endothelial dysfunction, which is the recognized preliminary stage for the development of atherosclerosis and its cardiovascular complications. In review, we focus on the most recent data on:

- Epidemiology and pathophysiology of cardiovascular risk factors and complications in patients with PSO/PSA;
- Molecular background of links between PSO and Ath development;
- Impact of traditional and new treatments on cardiovascular risk in PSO;
- Possibilities of early diagnosis, prevention strategies and effective cardiovascular treatment as well as novel therapeutic perspectives in PSO/PSA.

The PubMed database was searched for publications in English until August 2021 with the usage of MesH search terms: psoriasis and psoriatic arthritis, in combination with cardiovascular disease, atherosclerosis and each of the standard and new treatments described in the article. Further publications were searched manually from the reference list in the papers. Only full text articles were considered for the reference list.

## 2. Cardiovascular Risk Factors and Complications in PSO/PSA

PSO affects 2–3% of the adult population and 1.4% of children worldwide with significant geographical differentiation (reaching, e.g., 0.91% in the U.S. and 8.5% in Norway), and it is related, especially in its severe forms, to the higher prevalence of cardiovascular (CV) risk factors and complications and other comorbidities representing a wide spectrum of internal diseases [3,4,5,6,7]. 

The doubled risk of major adverse cardiovascular events (MACE) was described for the first time in 1973 in PSO patients who were compared to patients with other dermatological disorders [8]. This was further confirmed by meta-analysis including 14 cohort studies identifying increased CVD (cardiovascular disease) risk in patients with severe PSO, defined as requiring systemic therapy or hospitalization [9]. This risk was also related to the severity and duration of the skin disease. Cardiovascular mortality, myocardial infarctions (MI) incidence, and, to a lesser extent, strokes as well as chest pains related to angina, symptoms of peripheral artery disease and atrial fibrillation were all reported in the studies including psoriatic patients [10,11,12,13].

The increased CVD risk in patients with milder form of PSO has not been well proven and some inconsistences exist regarding definitions and classifications of mild, moderate and severe PSO in various studies, e.g., related to area of the affected skin, use of systemic therapy or the possibility of treatment in the outpatient settings. Moreover, patients with psoriasis treated with conventional systemic treatments (e.g., ciclosporin and acitretin) may develop hyperlipidemia, which may result in an increased CVD risk in those patients. As far as the impact of psoriasis severity on CVD risk is concerned, Ahlehoff et al. [12], in Danish population-based study, reported that the odds ratio of predicting cardiovascular mortality in severe psoriasis was very similar to that observed in diabetic patients (1.57 with 95% CI 1.27–1.94 for PSO vs. 1.59 with 95% CI 1.56–1.63 for diabetes). Assessments of psoriasis severity used in the studies and the relationship between traditional risk factors (dyslipidemia, family history, arterial hypertension, age, cigarette smoking, diabetes mellitus, obesity and physical inactivity) and psoriasis may all cause methodological problems hampering the accurate assessment of CV risk in PSO [8]. 

Beyond the “hard” and “soft” clinical endpoints, the prevalence of various indices of atherosclerosis progression or its consequences were assessed in numerous studies of PSO patients, finding the correlation between PSO and calcification of coronary arteries, higher prevalence of traditional risk factors, increased arterial stiffness and carotid intima-media thickness, as well as a connection between the femoral atherosclerotic plaques thickness and the duration of PSO [14,15,16,17]. Other studies found impairments in flow-mediated dilatation of arteries, increased epicardial adipose tissue measured by CT, MRI or transthoracic echocardiography or hypertrophy and deterioration of heart ventricular and atrial function in echocardiography [18,19,20,21,22].

Interestingly, large cohort analysis did not reveal any association with the short to medium-term risk for major CV events for psoriasis overall and severe psoriasis when adjusted for known CVD risk factors [23].

Nevertheless, endothelial dysfunction, being the starting point for Ath development, seems to be strongly related to various autoimmune and inflammatory diseases also in the absence of traditional CV risk factors [24]. The microvascular dysfunction itself can lead to increased number of CV complication and myocardial functional impairment even preceding full development of macroscopic atherosclerotic plaques [25,26]. Therefore, proinflammatory and pro-atherosclerotic phenotypical modulation of endothelium forms a crucial link between traditional and novel risk factors and atherosclerotic or sensu lato CV complications.

Moreover, in recent years, the evidence regarding increased cardiovascular mortality, which may also be described as a possible shortening of life expectancy of about 5–6 years in patients with severe PSO, has been continuously accumulating [27,28,29].

The main examples of comorbidities in psoriatic patients and their postulated pathophysiological links are presented in Table 1.

## 3. Molecular Background of Links between PSO and Ath Development

Immune-mediated inflammation of the skin is a key feature of PSO that can be modified by different genetic and environmental factors. Whereas the role of Th1 cells response driven by IFN-γ and IL-12 in PSO has been established for over three decades, recent developments added the importance of the chronic activation of IL-23 and Th17 axis, in which IL-23 maintains the activity of Th17 cells [85].

In 2011, Boehncke et al. [86] proposed and developed the concept of “psoriatic march” to describe how systemic psoriatic inflammation may lead to insulin resistance as well as endothelial cell dysfunction, causing atherosclerosis preceding cardiovascular complications. Although increased risk of CVD is present in various autoimmune diseases (such as rheumatoid arthritis or inflammatory bowel diseases), Ath and PSO also share a joint profile of inflammatory cells (T lymphocytes, monocytes and macrophages) as well as cytokines. This concept led to further development of the theory of “two plaques for one syndrome” which was supported by the evidence of a shared pattern of T helper cells and Th17 cytokine upregulation with increased expression of adhesion molecules and documented displacement of inflammatory cells between psoriatic skin lesions, peripheral circulation and atherosclerotic plaques driven by cytokines released from PSO lesions [87,88,89,90].

Furthermore, pro-inflammatory cytokines known as key factors for PSO lesions development, IFN-γ (interferon gamma), TNF-α (tumor necrosis factor alfa) and IL-17, were found in increased amounts in unstable atherosclerotic plaques in patients with acute coronary syndromes, whereas cardiovascular biomarkers such as MCP-1 (monocyte chemoattractant protein) as well as macrophage-derived chemokines were measured in skin and serum in PSO patients [91,92,93].

Similarly, patients with ischemic heart disease have higher levels of Th17-related cytokines (IL17, IL6 and IL8) circulating in their blood and there are data showing that overexpression of Th17 cytokines in PSO may mediate the development of Ath and cardiovascular comorbidities [94,95]. Moreover, also in patients with psoriatic arthritis (PSA), without significant burden of traditional cardiovascular risk factors or history of CVD, levels of pro-atherogenic inflammation markers such as CRP (C-reactive protein) and soluble ICAM-1 (intracellular adhesion molecule-1) when compared to healthy subjects were related to the duration and severity of PSA. This underlines the need for considering primary prevention measures in such patients [96]. Because of common inflammatory pathways (Th1 and Th17 cytokines, etc.) chronic skin inflammation in PSO may lead to vascular, subcutaneous and systemic inflammation, which may be confirmed by PET/CT scans in particular organs, e.g., aorta or liver, and may further result in Ath development and thrombotic complications [97,98,99].

Recently, closer attention has been paid to the role of activated inflammasomes in the pathogenesis of psoriasis, which are large protein complexes able to cleave the proinflammatory cytokines: pro-interleukin-1β and pro-interleukin-18 into active forms which contribute to PSO development [100,101,102].

Going beyond common phenotypic expression of systemic inflammation, there is an ongoing search for shared genetic background between PSO/PSA and Ath [103]. Joint genetic factors were postulated for PSO, type II diabetes and Crohn’s disease [48]. Other authors showed that polymorphism of apolipoprotein E (*APOE*) gene and common variation of *IL12B*, *IL23R* and *IL23A* genes may play a role in patients with PSO, PSA and DM type 2 [48,104,105].

Although psoriatic or wider “inflammatory skin march” theory assumes the causative role of skin inflammation causing the atherosclerosis and its thrombotic cardiovascular complications, the question of exact time relationship between PSO and Ath or “plaque precedency issue” remains unresolved since strong evidence on a higher risk of PSO development in patients with CV risk factors exist (see Table 1) [106].

**Figure 1:**
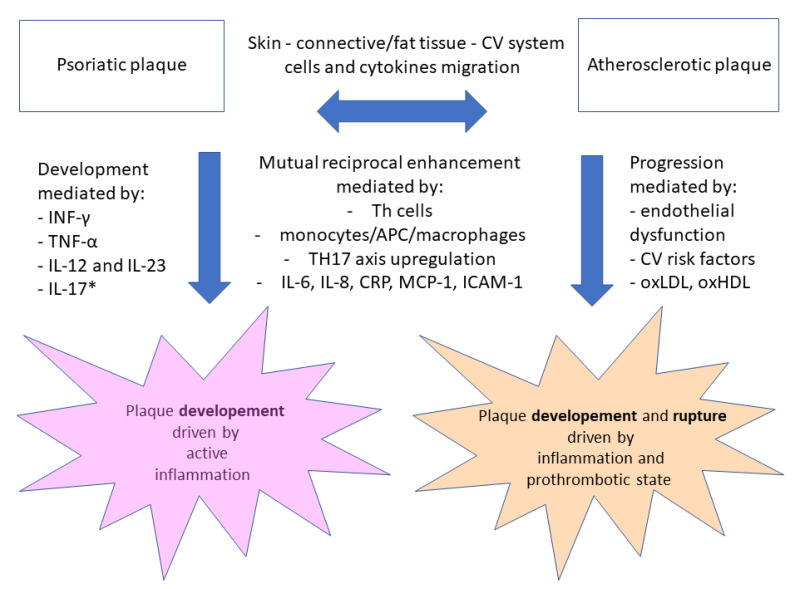
Interconnection between PSO and Ath—two plaques’ interactions. Notes: IL-12 and IL-2—interleukins sharing common p40 subunit, IL-17*—interleukin with potential pro- and anti-atherosclerotic activity in PSO plaque stimulates hyperproliferation and differentiation of keratinocytes, maturation of dendritic cells and recruitment of neutrophils and macrophages in psoriatic lesions. Abbreviations: CV—cardiovascular, INF-γ—interferon gamma, TNF-α—tumor necrosis factor alfa, IL—interleukin, APC—antigen presenting cell, CRP—C-reactive protein, MCP-1—monocyte chemoattractant protein-1, ICAM-1—intracellular adhesion molecule-1, oxLDL—oxidized low-density lipoprotein, oxHDL—oxidized high-density lipoprotein.

Present knowledge regarding two plaques’ interaction and the connection between fat tissue, cardiovascular and gastrointestinal system and their circulating mediators is illustrated in Figure 1.

An increasing amount of evidence supporting joint pathomechanisms in psoriasis severity and CV complications raise the question related to the possible beneficial impact of systemic anti-inflammatory treatment, applied with great success in recent decades in PSO/PSA patients, on lowering the prevalence of cardiovascular complications as well as cardiac and general mortality [107].

## 4. Impact of Traditional and New Treatment on Cardiovascular Risk in PSO

Traditional systemic drugs used in PSO/PSA treatment show varied impact on Ath progression and cardiac complications. Consistently detrimental impact on CVD and risk factor burden was observed for cyclosporine due to the possible development of dyslipidemia, hypertension, and impairment of renal function, which all should be limited by shortening the period of cyclosporine treatment [108]. Data related to acitretin influence on CV risks are conflicting. The increase in the risk of hypertension and dyslipidemia as well as CVD was described in acitretin treatment, but on the other hand, some beneficial effects on atherosclerotic plaques formation were also reported [109]. Beneficial changes in the profile of CV risk biomarkers with lowering CRP serum levels and increasing adiponectin resulting in the improvement of endothelial function were described for therapy with fumaric acid esters in patients with moderate to severe PSO [110]. The reduction of 21% in CVD and 18% in MI incidence was reported in meta-analysis in PSO patients treated with widely used oral or subcutaneous methotrexate [111].

Systemic biological therapies brought significant, short and long-term improvement in the PSO and PSA treatment, confirmed by the reduction in skin lesions severity, functional and QoL improvement as well as the reduction in hospitalization and lowering the number of rheumatological complications. High clinical efficacy of biological therapies blocking the activity of TNF-α and interleukins (12/23, 17) provided the “ex iuvantibus” evidence of the cytokine roles in the skin and joints’ psoriatic involvement. Less certain and with varied data is the impact of various cytokine inhibitors on the incidence of cardiovascular events, with more evidence gathered on the protective role of TNF-α compared to, e.g., IL 17 inhibitors, which have been used for a shorter time [112]. In Table 2, we present biologic medications frequently used nowadays in PSO/PSA treatment with their respective target of action and the connection with CVD.

Elevated levels of TNF-α and its soluble receptors present in severe PSO lead to the development of atherosclerosis, the deterioration of cardiac function and the remodeling of smooth muscles. Similarly, the treatment with TNF-α and IL12/23 inhibitors was reported to improve various “soft” endpoints in the cardiovascular system, including the reduction in aortic inflammation in PET assessment, slowing the calcification of coronaries in CT study or enhancing the myocardial left and right ventricular function in echocardiography [124,125,126,127].

Nevertheless, there are also data revealing the lack of improvement, as it was shown for mortality or hospitalization (etanercept) in a RENEWAL study in 1500 patients with heart failure, or even detrimental effects on mortality in patients treated with high doses of infliximab [128,129]. To sum the contemporary data, it may be stated that beyond patients with symptomatic heart failure, anti-TNF- α agents exert protective impacts on cardiovascular risk, similar for all three widely used in PSO/PSA medications (etanercept, infliximab and adalimumab), better when compared to patients treated only with topical therapy, phototherapy or methotrexate.

Interleukins 12 and 23 play an important role in the pathogenesis of inflammatory process both in psoriasis and atherosclerosis. Released by macrophages and dendritic cells, they activate Th1 and Th17 lymphocytes to produce other cytokines, including TNF-α and IL17 as well as IFN-γ, IL17F and IL22, whose role in PSO and Ath has been recently documented [130,131]. Moreover, both IL12 and 23 share common subunit p40, the lack of which in knocked-out laboratory mice cause resistance in developing autoimmune conditions, including psoriasis. Therefore, anti-IL12/23p40 biologic therapy was introduced with ustekinumab and briakinumab [132]. For both agents mentioned above, safety concerns related to the possible increase in CV events, especially in early phase of treatment, were raised, which then seemed to be effaced in the longer-term follow-up [119,133]. The pathophysiologic background for these clinical observations related to a temporary increase in pro-atherogenic mediators during the initial stage of therapy, which, however, significantly decreased further into the duration of the treatment [134]. Nonetheless, the usage of briakinumab was stopped and the exact impact of the inhibitors of IL12/23p40 needs further detailed analyses.

The studies and discovery of the increased activity of Th17 cells and IL 17 levels in patients with PSO were followed by the successful introduction of several anti-IL-17 agents to the treatment of psoriasis [135,136]. Anti-IL-17 monoclonal antibodies secukinumab, ixekizumab as well as brodalumab, being an anti-IL-17 receptor antibody, showed both good efficacy and safety in patients with moderate to severe PSO. However, since IL-17 has been shown to exhibit both pro- and anti-atherogenic effects depending on inflammatory background, the potential impact of the anti-IL17 drugs on CV complications is uncertain. Overall, in pooled analysis including 10 studies, the incidence of cardiovascular complications in patients with PSO treated with secukinumab was low and they were mostly recorded in patients with present CVD risk factors such as arterial hypertension and dyslipidemia [137]. Likewise, analysis of seven trials revealed similar or even better long-term CV safety profile of ixekizumab as compared to etanercept, which was also consistent with earlier reports on ixekizumab’s safety [138,139]. Reports on the beneficial effects of anti-IL-17 drugs in renal function are also available [140]. As far as brodalumab is concerned, present data are too sparse to draw conclusions about the impact of this drug on cardiovascular outcomes, since only some preliminary suggestions toward both protective and detrimental effects have been so far published [141,142]. 

Bimekizumab is a humanized monoclonal antibody, a dual inhibitor of interleukin IL-17A and IL-17F, which was recently approved in Europe for the treatment of moderate to severe plaque psoriasis in adults [143]. Clinical phase II trials already showed good efficacy in both psoriasis and psoriatic arthritis [144,145]. No major adverse cardiac events were reported in the published studies [143,144,145].

To summarize this part of the review, it seems that anti-TNF-α agents have a better confirmed role in diminishing CV complications in the short-term treatment of PSO, whereas the role of newer drugs remains, at this moment, conflicting [146].

## 5. Novel Diagnostic and Therapeutic Perspectives in PSO/PSA

Given the well-confirmed relationship between PSO, Ath and cardiovascular complications, the reduction in detrimental outcomes may be closely related to effective global risk stratifications and preventive treatment reaching beyond the pharmacotherapy limited to the skin and joints [147]. As the traditional scores based on classical risk factors underestimate the CV risk in PSO/PSA (similarly to autoimmune diseases such as lupus or rheumatoid arthritis), the reclassification to the higher risk group is needed for psoriatic patients (especially with PSA), as documented in the Framingham and other popular scores [148,149]. The proposed solution for an improvement in the risk assessment in patients with PSO was to apply 1.5 multiplier to calculated CV risk score in psoriatic patients or to add other tests revealing subclinical atherosclerosis, such as carotid ultrasound with intima-media thickness assessment, pulse wave velocity measurements or coronary calcium score evaluation [150].

Taking into account current European and North American guidelines regarding primary and secondary prevention of cardiovascular complications, the increased burden of CV events in PSO as well as data underlining the potential of statin therapy for reducing psoriasis severity, Masson et al. [6] recommended high-intensity statin treatment for secondary prevention in PSO patients with severe hypercholesterolemia (LDL-C > 190 mg/dL or familial) and high CV risk scores (calculated in patients with psoriasis with adjustment of 1.5 multiplier). Psoriatic patients with diabetes mellitus or subclinical atheromatosis should have a choice between starting moderate or high-intensity statin, whereas moderate-intensity statins were recommended for moderate CV risk scores and moderate to severe chronic kidney disease in those patients [6].

In the treatment of HTN, the long-term use of beta-blockers was associated with PSO development related to blocking beta receptors in the skin as well as increasing phosphorylation in T cells and favoring cellular proliferation and the release of cytokines [54,151]. Additionally, the exacerbation of PSO was also observed in patients taking angiotensin-converting enzyme inhibitors or angiotensin II receptor blockers [152]. As a potential result of these drug-related side-effects, patients with PSO achieved worse control of hypertension [37].

Contrarily, hypoglycemic agents such as GLP-1 agonists, DPP-4 inhibitors, thiazolidinediones and biguanides seem to reduce incidence of autoinflammatory and autoimmune disorders including PSO and alleviate the disease severity [41,153,154,155].

Novel systemic biologic drugs in PSO/PSA treatment include agents blocking IL12/23, IL-17 and, recently, IL-23p19 units. Recently published meta-analysis showed that IL-17 inhibitors were the most effective in achieving improvement in PSO severity (e.g., as reported with PASI score) at 12 weeks as compared to IL12/23 and IL-23 inhibitors [122]. On the other hand, ustekinumab (older type IL12/23 inhibitor blocking p40 common unit) had a moderate efficacy and safety assessment. Among the novel drugs, risankizumab is an IL-23 inhibitor with good efficacy and safety profile, probably better than those of IL-17 blocking agents [156].

Mirikizumab is currently at the stage of clinical trials (examined in OASIS-1/2/3 studies), showing, so far, non-inferiority at week 16 and superiority at week 52 as compared to secukinumab [157]. Preliminary results also seem to report a similar clinical effectiveness between new anti-IL-23 drugs and anti-IL-17 inhibitors [158].

Janus kinase (JAK) inhibitors belong to a relatively new class of small-molecule drugs [159]. So far, they have been registered in various countries for ulcerative colitis, rheumatoid arthritis, atopic dermatitis and psoriatic arthritis treatment [159]. Clinical trials in moderate to severe psoriasis are currently ongoing [160,161]. JAK inhibitors act inside the cells, blocking the pathway mediated by JAK and STAT (signal transducer activator of transcription) proteins, leading to inhibited gene transcription for inflammatory cytokines [159]. Oral route of administration and potentially lower cost are advantages of this group of medications [159]. Tofacitinib in combination with methotrexate is indicated for the treatment of active psoriatic arthritis [162]. In clinical trials for rheumatic arthritis and psoriasis, an increase in both HDL and LDL cholesterol levels as well as triglycerides was described during treatment with tofacitinib [163]. However, in the summary of product characteristics, those side effects were described as uncommon, while HTN was reported in 2.2% of patients [162]. Venous thromboembolism (VTE) in the form of pulmonary embolism and deep vein thrombosis have been described in patients taking tofacitinib; therefore, it should be used with caution in patients with risk factors for VTE in all indications and dosages [162,164]. At least four other JAK inhibitors are currently undergoing clinical trials in psoriasis [159].

In a recently published review by Piros et al. [165], the authors underline the need for holistic care of patients with severe PSO, which goes beyond skin and joints and by self-reinforcing Th1 and Th17 cell-dependent systemic inflammation, accelerating the progression of metabolic and cardiovascular diseases. Although the intrinsic anti-atherotic potential of novel systemic biologic treatment is still improving, their action should be accompanied, and for older agents, counterbalanced, by further safe and effective preventive measures.

## 6. Conclusions

PSO and Ath share common biochemical pathways, including oxidative stress, endothelial dysfunction, monocyte and neutrophil recruitment, T cell activation, increased angiogenesis and prothrombotic state.

To optimize care for PSO/PSA patients, the connection between inflammation and atherosclerosis should be kept in mind and the multidirectional aspect of systemic therapies should be considered. Together with significant progress in the biologic therapy respective screening methods, adjusted global cardiovascular risk assessment and additional tests revealing initial stages of vascular and organ damage should be advocated.

The repeated yearly evaluation and patient education on a healthy lifestyle and non-pharmacologic and pharmacologic risk factor management is recommended. The introduction of early and vigorous prevention measures may help to avoid significant lifespan shortening observed in moderate to severe PSO/PSA, depending on CV complications as well as the impairment of life quality.

## Figures and Tables

**Table 1:** Comorbidities in psoriatic patients and their postulated pathophysiological links.

Disease/State Related to Psoriasis	Pathophysiologic Link and Chosen References
Psoriatic arthritis (PSA)	This form of psoriasis (PSO) involving joints is related to higher prevalence of cardiovascular and metabolic risk factors, atherosclerosis progression, as well as cardiovascular complications including myocardial infarction and strokes as compared to psoriasis limited only to the skin [30,31,32,33].
Metabolic syndrome	Increased prevalence of traditional risk factors: arterial hypertension (HTN), dyslipidemia (abnormalities found even in pediatrics populations as well as data suggesting increased risk of PSO development in long-lasting dyslipidemia), obesity, diabetes mellitus and their clustering in psoriatic patients [34,35,36], evidence of more difficult control of risk factors in psoriatic population [37], impaired cholesterol efflux capacity from macrophages observed in psoriasis because of higher level of oxidized high-density lipoprotein (oxHDL) [38], higher lipoprotein (a), apolipoprotein B and oxidized low-density lipoprotein (oxLDL) levels [39], as well as increased epicardial fat amount (expressed as thickness or area) [40]. Well-controlled glycaemia in diabetics was related to better control of skin psoriasis [41].
Nonalcoholic fatty liver disease	High triglycerides and abnormal serum fatty acid profile were also described in PSO [42]. In obese psoriatic patients treated with anti-TNF-alpha medications, dose–response relationship and particular tendency to central obesity were described. Beneficial effect of weight loss on improvement of PSO, including the increase in the efficacy of the treatment, was also observed [43,44,45,46].
Inflammatory bowel disease (IBD): Crohn’s disease and ulcerative colitis	Increased levels of IL-17 in both IBD (both in serum and bowel mucosa) and PSO [47]. Common pleiotropic susceptibility loci identified in PSO, type II diabetes and Crohn’s disease (genes related to locus 6p21 coding the major histocompatibility complex—MHC, genes related to IL23R and IL12B, the latter encoding p40 subunit essential in pathogenesis of IBD and PSO), shared clinical course and immunologic features [48,49,50]. Postulated similar abnormalities of gut microbiota and role of gut–skin axis in IBD and PSO/PSA, characterized generally by decreased bacterial diversity of gut microbiota beyond skin dysbiosis typical for PSO [51,52,53]. Increased risk of Crohn’s disease and ulcerative colitis in PSO as well as in PSA [54].
Cardiovascular disorders	Reciprocal relationship between the increased prevalence of HTN in psoriasis as well as more frequent development of PSO in hypertensives observed, e.g., in a Nurses’ Health Study—the use of beta-blockers for HTN treatment was postulated as a potential mechanism [55]. The amount of adipose tissue which in PSO is increased also around vessel walls is the source of angiotensinogen, converted then to angiotensin II, which further stimulates Th17 cells and enhanced hypertension by vascular dysfunction [56]. Increased cigarette smoking and alcohol use reported in PSO as well as an association between smoking and PSO severity. The increased amount of Th17 cells in blood in psoriatic patients who smoke was observed [57,58]. Evidence for increased number of myocardial infarctions and strokes as well as CV mortality in PSO, PSA, as well as rheumatoid arthritis patients [59,60,61]. Some studies indicated the increased number of venous thrombosis (VTE) in PSO patients. This trend was confirmed by meta-analysis although without reaching statistical significance [62,63,64,65,66]. Augmented risk of new-onset heart failure showing severity-dependence in psoriatic patients was observed in a Danish nationwide cohort study [67]. Increased prevalence of dilated cardiomyopathy was observed in PSO [68].
Lymphoma and other malignancies	PSO seem to be related to an increased risk of cancer, especially keratinocyte cancers and lymphomas, including cutaneous T-cell lymphomas (CTCL) [69,70,71,72]. PSO treated with psoralen-UV-A (PUVA) therapy shows highly increased risk of squamous cell carcinomas [73]. The increased risk of CTCL in PSO may be explained by persistent immune activation leading to the development of a dominant clone [74]. Certain known risk factors for cancerogenesis (such as alcohol and smoking) are more prevalent in psoriatic patients and some studies postulated the increased risk of lung, bladder, breast (observed in PSA patients in 3 studies) and colorectal cancer in psoriatic patients [75,76] as well as an increased risk of overall cancers after adjustment for confounders may be connected to this phenomenon [77]. The impact of conventional vs biologic treatment as well as potential differences in patients with PSA needs further analysis.
Depression	Chronic inflammation and endothelial dysfunction as well as higher incidence of traditional risk factors in PSO; additionally, sleep and anxiety disorders as well as depression led to further increases in CV complications [78,79,80]. Impaired quality of life, typical for chronic, systemic inflammatory disease, as well as for significant skin and joint involvement [81,82]. Observed reciprocal association between decreased probability of performing more intense physical activity when suffering from PSO, as well as reduced risk of PSO incidence in those performing vigorous physical activity [83,84].

**Table 2:** Biologic medications used in PSO/PSA treatment with their respective target of action and the connection with CVD.

Group of Medication	Name of Drug	Specific Targets and Actions in Immune System/Role in Pathogenesis and Treatment
TNF-α inhibitors	Infliximab Etanercept Adalimumab Certolizumab-pegol Golimumab	Target elevated levels of TNF-α; cause CRP, VEGF and resistin as well as chemotactics factors’ reduction: VCAM-1, E-selectin, IL-8, MCP-1 [113]. Additionally, decreases Th17 cell count in blood by stopping CD4+ T cells differentiation into Th1, Th17 and Th22. Improvement of insulin sensitivity [114] and showing antiplatelet activity, especially in patients with increased platelet activation prior to treatment, as evidenced by decreases in P-selectin levels [115] (data for TNF inhibitors and ustekinumab). Reducing level of circulating retinol-binding protein 4: RBP4 (showed to be linked to subclinical atherosclerosis with positive correlation with intima-media thickness) [116]. Adalimumab therapy improved pulse wave velocity (being strong predictor of CV events) after 6 months of therapy [117].
Inhibitors of the p40 subunit to IL-12 and IL-23	Ustekinumab Briakinumab	Human monoclonal antibodies from this group showed improvement of myocardial and vascular function as compared to treatment with cyclosporine or TNF alfa inhibitors [118]. During the initial stage, the temporary increase in pro-atherogenic mediators was observed. Small, randomized trials showed the increase in CV events with briakinumab (but not with ustekinumab), which was then withdrawn from application for psoriasis treatment [119,120].
IL-17A inhibitors	Secukinumab Ixekizumab	Confirmed high, dose-dependent efficacy in improving PSO during short-term 12–16-week treatment [121,122]. Moreover, secukinumab improved myocardial and vascular function as assessed by global longitudinal strain and pulse wave velocity measurements, respectively, which achieved significance in comparison to MTX and cyclosporine therapy after 12 months of follow-up [123].
IL-17 receptor antagonist	Brodalumab	
Novel inhibitors of IL-23 (blocking p19 unit of IL23)	Guselkumab Risankizumab Tildrakizumab Mirikizumab	Data are still being gathered; risankizumab being a new type of IL-23 inhibitor, seems to present better short-term efficacy [122]. In comparison with IL-17 antagonists, the novel group seems to show a better safety profile [122].

## Data Availability

Not applicable.
